# Caspase-8-and Gasdermin D (GSDMD)-Dependent PANoptosis Participate in the Seasonal Atrophy of Scented Glands in Male Muskrats

**DOI:** 10.3390/ani14223194

**Published:** 2024-11-07

**Authors:** Xiaofeng Tong, Xuefei Zhao, Yue Ma, Haimeng Li, Jinpeng Zhang, Zuoyang Zhang, Sirui Hua, Bo Li, Wei Zhang, Yu Zhang, Suying Bai

**Affiliations:** 1 College of Wildlife and Protected Area, Northeast Forestry University, Harbin 150040, China; 2021125336@nefu.edu.cn (X.T.); mayue@nefu.edu.cn (Y.M.); luoricizhui@163.com (J.Z.); zghnzzy123@163.com (Z.Z.);; 2 Detecting Center of Wildlife, State Forestry and Grassland Administration, Harbin 150040, China; 3 National Forestry and Grassland Administration Research Center of Engineering Technology for Wildlife Conservation and Utilization, Harbin 150040, China

**Keywords:** muskrat, scented gland, apoptosis, pyroptosis

## Abstract

The muskrat (*Ondatra zibethicus*) is an animal with special economic significance whose scented glands rapidly atrophy during the non-breeding season, but the mechanism of atrophy is not clear, with significant differences in apoptotic and pyroptotic signaling pathway expression according to transcriptome sequencing. During the non-breeding season, key apoptosis-related genes such as *Tnfr1* (TNF Receptor Superfamily Member 1A), *TRADD* (TNFRSF1A Associated via Death Domain), *FADD* (Fas Associated via Death Domain), *Casp-8* (Cysteine-aspartic proteases-8), and *Bax* (Bcl-associated X protein) were upregulated in the scented glands, while *Bcl2* (B-cell lymphoma-2) expression was downregulated. In the classical pyroptosis pathway, the mRNA expression levels of key genes including *Nlrp3* (the Nod-like receptor family pyrin domain-containing 3), *ASC* (the apoptosis-associated speck-like protein), *Casp-1* (Cysteine-aspartic proteases-1), *Gsdmd* (Gasdermin D), and *IL-1β* (Interleukin 1 Beta) were higher during the non-breeding season, similar to the transcription level of *Ripk1* (Receptor Interacting Serine/Threonine Kinase 1) in the non-canonical pyroptosis pathway, while *TAK1* (transforming growth factor kinase) expression was downregulated in this latter pathway. TUNEL assays and immunofluorescence analysis indicated increased apoptosis and GSDMD and Caspase-8 protein levels during the non-breeding season. Indeed, the protein levels of GSDMD-N, Caspase-8 p43, and Caspase-8 p18 were significantly higher during the non-breeding season, while the GSDMD levels were significantly lower compared to the secretion season. These results suggest that apoptosis and pyroptosis play regulatory roles in scented gland atrophy and that there is an interplay between them during this process.

## Simple Summary

Muskrats’ scented glands atrophy with the arrival of non-breeding periods, but the underlying mechanism is still unclear. Programmed cell death (PCD) is an important pathway of cell death that is closely related to the stability of the intracellular environment and the occurrence of diseases. Our research indicates that the expression levels of various apoptosis-and pyroptosis-related genes increase during the atrophy of muskrats’ scented glands. Therefore, we believe that apoptosis and pyroptosis may be involved in the rapid atrophy of muskrats’ scented glands during the non-breeding period.

## 1. Introduction

The muskrat (*Ondatra zibethicus*) is a semi-aquatic mammal of significant economic and ecological value, widely distributed across the northern hemisphere [1]. In northeast China, muskrats enter their breeding season between late April and early May, lasting until the end of August, followed by the start of a non-breeding season in September. Male muskrats have a pair of glands located between the skin and muscles of their abdomen, which rapidly develop and enlarge during the breeding season to secrete muskrat musk and then quickly shrink and lose their ability to synthesize and secrete this smell during the non-breeding period [2].

There has been relatively little research on the developmental physiology of muskrats. Due to the high correlation between the cyclical changes in muskrats’ scented gland development and their reproductive cycle, it is reasonable to speculate that the regulation of gland development is closely related to the levels of sex hormones. Various studies have supported this idea that sex hormones directly or indirectly influence the development of muskrats’ scented glands. Li et al. [3] found that muskrats’ scented glands can synthesize androgens, which could then regulate the seasonal differences in gland development through endocrine, autocrine, or paracrine mechanisms. Additionally, research by Cao et al. [4] and Zhang et al. [5] demonstrated that prolactin (PRL), the follicle-stimulating hormone (FSH), and the luteinizing hormone (LH) likely play roles in muskrats’ scented glands’ seasonal development by measuring changes in their blood levels and the expression patterns of their receptors. The musk glands’ seasonal development and musk secretion are regulated by the testes [6]. However, due to the lack of in-depth studies, the detailed molecular mechanisms underlying the regulation of muskrats’ scented glands remain unclear.

Programmed cell death (PCD) is a type of cell death that is actively and orderly controlled by genes, playing an essential role in normal tissue development and the elimination of aging or damaged cells [7], with common forms including apoptosis, pyroptosis, ferroptosis, necroptosis, and autophagy [8,9,10,11]. Recently, a new concept has been proposed to collectively refer to pyroptosis, apoptosis, and necroptosis as pan apoptosis, which can further describe the crosstalk between different cell death pathways [12]. By jointly activating cell pyroptosis, apoptosis, and necroptosis, inflammatory cells will be eliminated. Therefore, pan apoptosis can avoid pathogen-mediated death escape [13,14]. Of these, apoptosis is the best understood at the molecular level, characterized by apoptotic body formation and DNA fragmentation, but, due to the preservation of cell membrane integrity, it does not trigger a strong inflammatory response [15,16,17]. While there are numerous pathways and signals inducing apoptosis, the TNFR1 and Fas-driven ones are the typical extrinsic apoptosis pathways, both involving Caspase-8 activation, which subsequently activates downstream caspase family members to execute the apoptotic program [18,19,20,21,22,23].

Pyroptosis is a recently identified form of PCD, distinguished by continuous cell swelling until the membrane ruptures, releasing the cellular contents and triggering a strong inflammatory response [24], while, initially, apoptosis and pyroptosis were considered independent forms of PCD, growing evidence indicates that there is substantial overlap and crosstalk between its various mechanisms [25].

The Gasdermin (GSDM) family comprises key effector pyroptosis proteins, first reported by Ding, J et al. [26], who revealed the effector mechanism of GSDMD: upon cleavage by inflammatory caspases, the GSDMD’s N-terminal domain is released, possibly disrupting cell membrane integrity. The NLRP3 inflammasome can be triggered by damage- and pathogen-associated molecular patterns (DAMPs and PAMPs, respectively). Once activated, NLRP3 binds with the ASC, and the NLRP3/ASC complex induces Caspase-1 activation, leading to the production of mature IL-1β and the cleavage of GSDMD, initiating classical pyroptosis [27].

Recent studies suggest that Caspase-8 can also directly cleave GSDMD via TNFR activation GSDMD, a process which is highly associated with TNFR-induced pyroptosis but operates independently of the inflammasome [28,29,30]. The rapid atrophy of muskrats’ scented glands during the breeding and non-breeding seasons is closely associated with PCD processes. This study aims to investigate the molecular mechanisms underlying the atrophy of muskrats’ scented glands from the perspective of PCD.

## 2. Materials and Methods

### 2.1. Ethical Statement

All the animals used in this study were treated in accordance with the guidelines of China’s national animal welfare legislation. The care and use of experimental animals were approved by the Animal Ethics Committee of Northeast Forestry University.

### 2.2. Animals

The muskrats used in this study were purchased from the Wuchang Muskrat Breeding Farm in the Heilongjiang province, China (126.33° E, 44.04° N). Samples were collected in early June (breeding season) and late October (non-breeding season). Muskrats were all 1-year-old males, weighing 800 ± 50 g. Upon arrival at the laboratory, the experimental animals were allowed free access to food (Huake, Shandong, China) and water for 3 days to reduce stress. After sample collection, the animals were euthanized following anesthesia. Muskrats’ scented gland tissues were flash-frozen in liquid nitrogen for total RNA, total protein extraction, and RNA-Seq analyses. The remaining gland samples were fixed in 4% paraformaldehyde.

### 2.3. RNA Isolation and RNA-Seq

Total RNA was extracted using the GeneJET RNA Purification Kit (Thermo Fisher, Waltham, MA, USA, K0731). cDNA synthesis was performed with the PrimeScript RT Reagent Kit with a gDNA Eraser (Takara, Shiga, Japan), following the manufacturer’s instructions. Muskrats’ scented gland samples from breeding and non-breeding seasons were used for RNA-Seq analysis (*n* = 3). Library preparation and sequencing on the Illumina platform (San Diego, CA, USA) were performed by Novogene Bioinformatics Technology Co., Ltd. (Beijing, China). The quality control of the raw reads was conducted using the Fastx toolkit software (https://hannonlab.schl.edu/fastx_toolkit/, accessed on 29 October 2023). To ensure that the number of fragments truly reflected the level of gene expression, the number of fragment reads was normalized to the number of mapped reads in the sample. The Fragments Per Kilobase Million (FPKM) value was used as an index to measure the level of gene expression. Differentially expressed genes (DEGs) between groups were identified by FDR < 0.05 and | log2foldChange| > 1, and GOseq and the Cluster Profiler were used for the gene ontology (GO) and Kyoto Encyclopedia of Genes and Genomes (KEGG) pathway analyses of significantly differentially expressed genes.

### 2.4. Quantitative Real-Time PCR

qPCR reactions were conducted using SsoAdvanced™ Universal SYBR^®®^ Green Supermix (Bio-Rad, 1725271, Hercules, CA, USA) on a CFX384 Touch Real-Time PCR Detection System (Bio-Rad). The qPCR reaction setup and cycling conditions were carried out according to the manufacturer’s protocols. Fluorescence signals were collected during the annealing/extension phase, and the melt curve analysis was performed using the default program. Actb was used as the normalization control, and the relative expression levels were calculated using the 2^−ΔΔCt^ method. The primer sequences are listed in Table S1.

### 2.5. Immunofluorescence Staining

The scented gland tissue samples were fixed with 4% paraformaldehyde and then gradient-replaced with methanol and PBST, followed by replacement with 5, 10, and 20% sucrose gradients. After soaking the samples overnight in a sucrose and OCT solution at a 1:2 ratio (G6059, Servicebio, Wuhan, China), the tissue samples were sectioned into 10 µm slices using a cryostat (Leica, CM1950, Solms, Germany). The sections were blocked at room temperature with BSA for 1 h and then incubated overnight at 4 °C with the following primary antibodies: Cleaved GSDMD (N Terminal) Rabbit mAb (Abclonal, A22523, Boston, MA, USA) and Caspase 8/p43/p18 Monoclonal antibody (Proteintech, 66093-1-Ig, Wuhan, China). The sections were then incubated at room temperature for 1 h with the following secondary antibodies: Goat Anti-Rabbit IgG (H + L) Fluor488-conjugated (affinity, #S001, Melbourne, Australia) and Goat Anti-Mouse IgG (H + L) CY3-conjugated (affinity, #S0012). The slides were mounted using an anti-fluorescence quenching mounting medium containing DAPI (G1407, Servicebio) and observed using a laser scanning confocal microscope (OLYMPUS, FV3000, Tokyo, Japan) to visualize the fluorescence signals.

### 2.6. Protein Isolation and Western Blotting

We extracted the proteins using the RIPA lysis buffer (Biosharp, Anhui, China) containing 10% PMSF. We measured the protein concentration using the BCA assay (Takara, T9300A) and then denatured the proteins by heating them at 99 °C for 10 min. We adjusted the sample volume to 20 μg and separated the proteins using 4–20% gradient SDS-PAGE gel (GenScript, M00653, Piscataway, NJ, USA) at 150 V for 40 min. Afterwards, we transferred the proteins to a PVDF membrane (ISEQ00010, Merck, Darmstadt, Germany), blocked them with 5% BSA at room temperature for 2 h, and then incubated them in a primary antibody overnight at 4 °C. Afterward, we incubated the above with the secondary antibodies at room temperature for 1 h. We then carried out the detection step using the ECL method, normalized the results using β-ACTIN, and analyzed the grayscale images with ImageJ software (Bethesda, MD, USA).

### 2.7. Terminal Deoxynucleotidyl Transferase dUTP Nick End Labeling

The terminal deoxynucleotidyl transferase dUTP nick end labeling (TUNEL) assay was used to detect apoptosis in the scented gland cells of muskrats, following the protocol provided with the TUNEL assay kit (Servicebio, G1504). Briefly, we fixed frozen sections of muskrats’ scented glands from both breeding and non-breeding seasons in a 4% paraformaldehyde solution (dissolved in PBS) for 15 min at room temperature. Then, we added 100 μL of Proteinase K working solution (20 μg/mL) to cover each sample completely and incubated them at room temperature for 10 min. We added an equilibration buffer to cover the entire area of each sample and incubated them at room temperature for 10 min. Afterwards, we added the TdT incubation buffer to each tissue sample, incubated them at 37 °C for 1 h, and then mounted them with DAPI-containing antifade mounting medium. We observed the fluorescent labeling under a fluorescence microscope (OLYMPUS, BX43, Japan).

### 2.8. Statistical Analysis

Statistical analyses were performed using GraphPad Prism 9.0 (San Diego, CA, USA). Data are expressed as the means ± SD. Statistical significance was assessed using Student’s *t*-test.

## 3. Results

**Figure 1:**
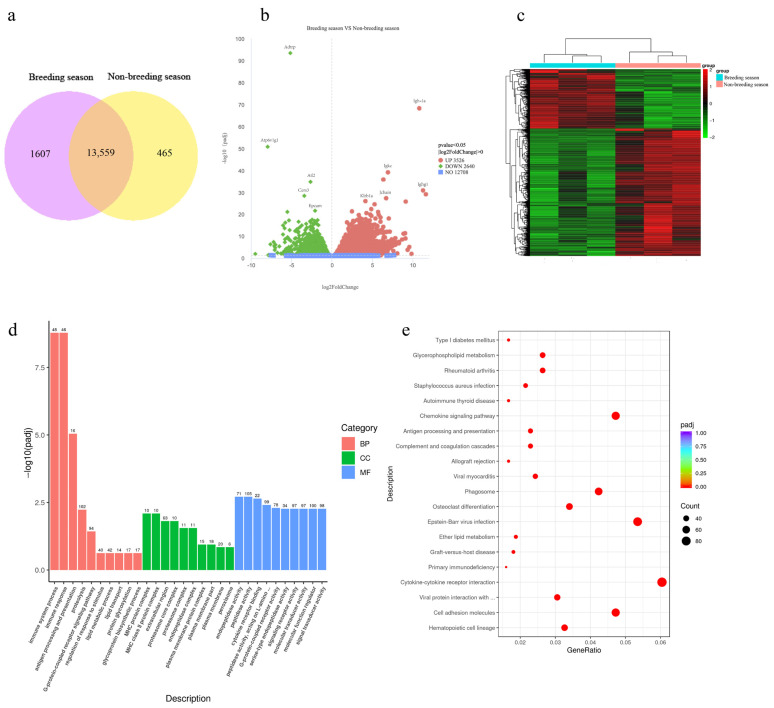
Transcriptomic analyses of scented glands during breeding and non-breeding seasons. (**a**) Venn diagram of shared and unique transcripts. (**b**,**c**) Clustering analysis heatmap and volcano plot of the differentially expressed genes. (**d**,**e**) GO and KEGG enrichment analyses.

The RNA-Seq results revealed a total of 15,631 transcripts in the muskrats’ scented gland tissues from both the breeding and non-breeding seasons, with 13,559 transcripts shared between the two groups (Figure 1a). The results refer to the CNGB sequence archive annotation (https://db.cngb.org/cnsa/, number CNP 0003335, accessed on 29 October 2023). The average RNA integrity number (RIN) is greater than 8.0 and 6he average RNA concentration is 6.25 μg. A total of 3440 differentially expressed transcripts were identified, with 2347 upregulated and 1093 downregulated during the breeding season compared to the non-breeding season. The clustering analysis of the differentially expressed transcripts is shown in Figure 1b, while the visualization of significantly differentially expressed genes is presented in the following volcano plot (Figure 1c). The GO analysis of the significantly differentially expressed genes (Figure 1d) indicated significant enrichment related to immune system processes, the immune response, and the regulation of programmed cell death and apoptotic processes. The KEGG pathway analysis (Figure 1e) revealed significant enrichment in pathways such as viral protein interactions with cytokine and its receptors, cytokine–cytokine receptor interactions, and the chemokine and NOD-like receptor signaling pathways.

The RNA-Seq results indicate that muskrats’ scented gland tissues are regulated by multiple mechanisms during the non-breeding season, involving extensive cellular signaling transduction. Notably, the differential expression and enrichment analysis highlighted significant changes in genes such as *trp*, *Nlrp1*, *Nlrp3*, *Aim2*, *Gsdmd*, *Casp-1*, *IL-1β*, and *pro-IL-1β*, suggesting that both apoptosis and pyroptosis may play crucial roles in the seasonal regulation of muskrats’ scented gland tissues.

**Figure 2:**
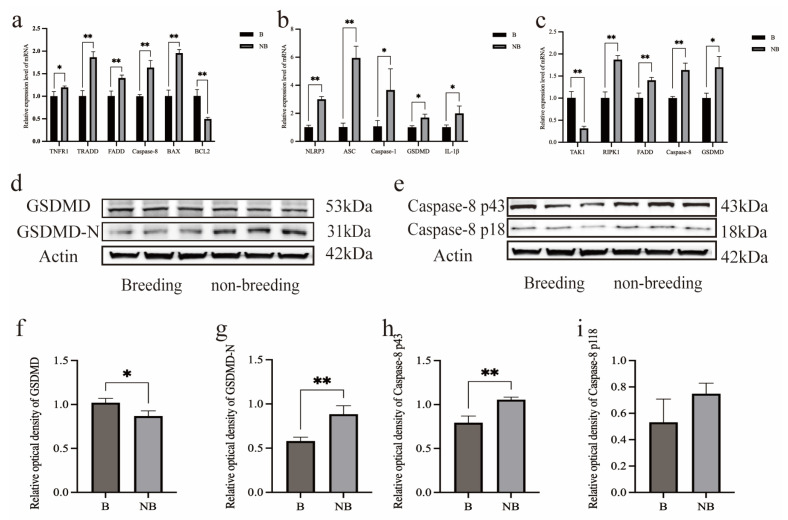
Real-time quantitative PCR was used to detect the mRNA expression levels of the following genes in muskrats’ scented glands during the breeding and non-breeding seasons. (**a**) TNFR1, TRADD, FADD, Caspase-8, BAX, and BCL2. (**b**) NLRP3, ASC, Caspase-1, GSDMD, and IL-1β. (**c**) TAK1, RIPK1, Caspase-8, GSDMD, and FADD. (**d**,**e**) Also shown are the protein expression results of GSDMD and Caspase-8. (**f**–**i**) The grayscale analysis of GSDMD, GSDMD-N, Caspase-8 p18, and Caspase-8 p43. B—breeding season; NB—non-breeding season. The error bars represent the means ± SEM (*n* = 3, each stage). * Statistical significance (* *p* < 0.05; ** *p* < 0.01).

The relative mRNA expression levels of *Tnfr1*, *TRADD*, *FADD*, *Casp-8*, *Bax*, *Nlrp3*, *ASC*, *Casp-1*, *Gsdmd*, *IL-1β*, *TAK1*, *Ripk1*, and *FADD* were compared between the breeding and non-breeding seasons using quantitative real-time PCR (Figure 2a–c). The breeding season was used as the control group, with its transcription levels set to 1. In the apoptosis pathway, the mRNA transcription levels of *Tnfr1*, *TRADD*, *FADD*, *Casp-8*, and *Bax* in the scented glands during the non-breeding season were significantly higher compared to the control (*p* < 0.01, *p* < 0.05) (Figure 2a), while the transcription level of *Bcl2* was significantly lower (*p* < 0.01). As the mRNA transcription level of *Bcl2* decreased, the mRNA transcription level of *Bax* increased, leading to a decrease in the *Bcl2*/*Bax* ratio.

The results of the mRNA level analysis for the classical pyroptosis pathway, including *Nlrp3*, *ASC*, *Casp-1*, *Gsdmd*, and *IL-1β*, can be found in Figure 2b. Compared to the control, the transcription levels of the *Nlrp3*, *ASC*, *Casp-1*, *Gsdmd*, and *IL-1β* genes in the scented glands were significantly higher during the non-breeding season (*p* < 0.01, *p* < 0.05). Then, the mRNA expression levels of the genes involved in the non-canonical pyroptosis pathway were also measured in the muskrats’ scented glands during both seasons: the expression of the *TAK1*, *Ripk1*, *Casp-8*, *Gsdmd*, and *FADD* genes showed significant differences compared to the control group (Figure 2c), with *TAK1* significantly downregulated (*p* < 0.01) and the rest significantly upregulated (*p* < 0.01, *p* < 0.05) in the non-breeding season (*p* < 0.01).

Western blot analysis was used to examine the seasonal protein expression of GSDMD and Caspase-8 in the scented glands. The results were normalized to the β-ACTIN expression levels. The main bands for GSDMD were observed at approximately 53 kDa, with the GSDMD-N fragment at 31 kDa (Figure 2d), while the Caspase-8 bands were detected at approximately 43 and 18 kDa (Figure 2e). The ImageJ software was used for the grayscale analysis (Figure 2f–i). The protein levels of GSDMD-N were significantly higher in the non-breeding season (*p* < 0.01), whereas that of GSDMD was lower compared to the control (*p* < 0.05), indicating that this protein is cleaved and active during the non-breeding season. Additionally, the Caspase-8 p43 levels were higher in the non-breeding season compared to the control (*p* < 0.01), similarly to Caspase-8 p18.

**Figure 3:**
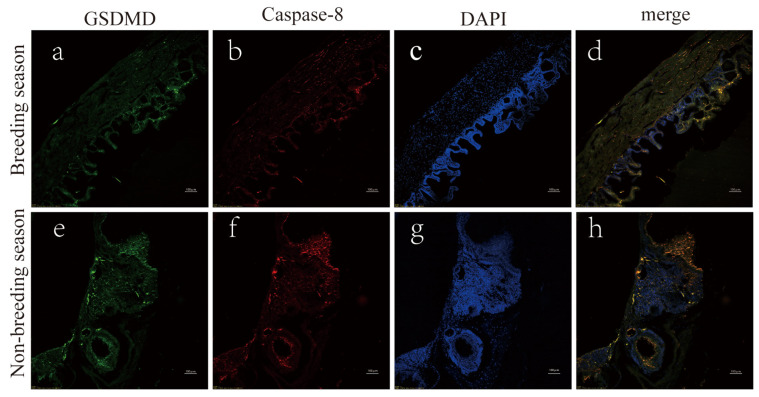
Immunofluorescence results in muskrats’ scented glands during the breeding (**a**–**d**) and non-breeding seasons (**e**–**h**). The green (**a**,**e**) and red (**b**,**f**) fluorescence signals represent GSDMD and Caspase-8, respectively. Scale bar = 100 μm.

**Figure 4:**
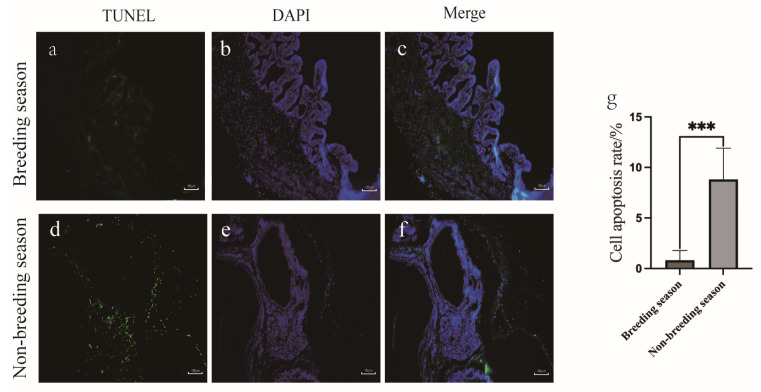
TUNEL results in scented glands during the breeding (**a**–**c**) and non-breeding seasons (**d**–**f**); scale bar = 100 μm. Apoptosis index during the breeding and non-breeding seasons is shown (**g**). The error bars represent the means ± SEM (*n* = 5, each stage). * Statistical significance (*** *p* < 0.001).

The immunofluorescence results showed that the scented gland tissue in muskrats during the breeding season (Figure 3a–d) was denser compared to the non-breeding period (Figure 3e–h). However, during the latter, the expression levels of Caspase-8 and GSDMD were higher. The TUNEL staining method was used to assess the seasonal impact on apoptosis in muskrats’ scented gland cells. The results (Figure 4) show that apoptotic cells were rare during the breeding season, whereas their number was significantly higher in the non-breeding season. The significant upregulation of the apoptosis index during the non-breeding season was observed (Figure 4g).

## 4. Discussion

Muskrats are seasonal breeders, and changes in their scented glands may be linked to their reproductive cycle. Rodents belong to seasonal reproductive animals, and this reproductive rhythm is predominantly regulated by factors such as the photoperiod, temperature, and food. Seasonal breeders develop gonads and display reproductive behavior at specific times of the year, restricting the birth of offspring to the period between spring and early summer [31,32]. Muskrats’ scented glands shrink during the non-breeding season and are replaced by a translucent fluid. Seasonal morphological differences in the scented glands were noted. The sequencing results revealed that, during the non-breeding season, the apoptosis levels were elevated, alongside those of many genes related to the pyroptosis pathway. A study by Li et al. [33] suggests that the degeneration of musk glands is associated with multiple signaling pathways, such as the calcium signaling pathway and the TGF beta signaling pathway, which are involved in processes such as cell apoptosis, differentiation, and proliferation, suggesting that the scented glands’ rapid shrinkage is regulated by both apoptosis and pyroptosis. The research of Zhang et al. [6] and Xie et al. [34] focuses on the effects of estrogen and androgen on the development of sebaceous glands. Similarly, during the non-breeding season, the level of glycerophoripid metabolism is significantly elevated; it participates in the structure and function of cell membranes and plays an important role in signal transduction, cell recognition, and immune response.

The extrinsic apoptotic pathway is typically mediated by death-inducing receptors such as Fas and TNFR, which bind to FADD proteins through their intracellular domains, in turn recruiting and activating *Casp-8* [35]. *Casp-8*, an initiator caspase, activates itself through autolytic cleavage and subsequently cleaves and activates downstream effector caspases, such as Caspase-3, ultimately triggering apoptosis [36].

In this study, our qPCR analysis revealed that the mRNA expression levels of *Tnfr1*, *TRADD*, and *FADD* in the scented glands during the non-breeding season were higher than the controls, indicating an enhanced role of the death receptor pathway in glandular cell apoptosis. The TUNEL assay results further confirmed this, showing a significant increase in the apoptosis levels in the scented gland cells during the non-breeding season.

The *Bcl2* expression level was significantly lower during the non-breeding season (*p* < 0.01), while the mRNA expression level of *Bax* increased significantly (*p* < 0.01), leading to a reduced *Bcl2*/*Bax* ratio. This suggests that, during scented gland atrophy, the *Bax* gene promotes apoptosis by inhibiting the *Bcl2* gene’s cell survival effects, resulting in glandular atrophy [37]. In this study, the relative expression level of the *Casp-8* gene was upregulated during the non-breeding season, with significant differences observed (*p* < 0.01). This result was also validated by Western blotting and immunofluorescence analyses, indicating that *Casp-8* may play a crucial regulatory role in scented gland atrophy.

Caspase-1-mediated pyroptosis is characterized by sustained cell swelling, until the cell membrane ruptures, leading to the release of the cellular contents and subsequently activating a strong inflammatory and immune response [38]. Endogenous danger signals can trigger the activation of the NLRP3 inflammasome [39], and pyroptosis is crucial for controlling microbial infections by inducing the production of inflammatory cytokines, subsequently causing intense inflammation [40]. During NLRP3 inflammasome assembly, the NLRP3 receptor protein interacts with ASC, which then recruits and binds to Caspase-1 to exert its effects. GSDMD is a critical substrate of inflammasome-associated caspases and serves as a key executor of pyroptosis. Mature Caspase-1 cleaves GSDMD to produce an approximately 31 kDa N-terminal fragment, which initiates pyroptosis and induces the secretion of mature inflammatory cytokines, meaning that GSDMD cleavage is sufficient to drive pyroptosis [24]. In this study, the transcription level of GSDMD was significantly higher during the non-breeding season compared to the breeding period, with similar results obtained through Western blotting for GSDMD-N protein expression and stronger GSDMD immunofluorescence signals observed during this period as well.

Pyroptosis primarily relies on the inflammasome activation of caspase family proteins to trigger various physiological responses. Our results indicated that the mRNA expression levels of *Nlrp3*, *ASC*, *Casp-1*, *Gsdmd*, and *IL-1β* in the scented gland during the non-breeding season were significantly higher compared to the breeding season, suggesting that the pyroptosis pathway may play a regulatory role in scented gland atrophy. Since the aberrant activation of PCD can lead to inflammation and pathological responses, regulating PCD pathways is crucial to maintaining the stability of the internal environment, with caspases being key regulatory proteins. Caspase-8 is one of the most universal caspases, playing an important role in regulating infection and inflammation [41], and is involved not only in apoptosis but also in pyroptosis [30].

**Figure 5:**
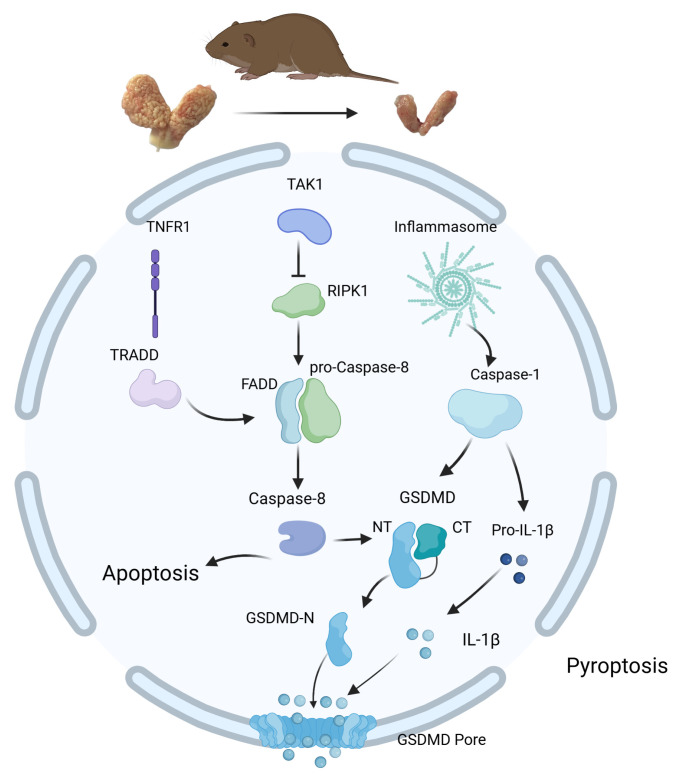
Sketch of apoptosis and pyroptosis involved in muskrats’ scented glands.

Caspase-8 can, under certain conditions, directly cleave GSDMD or activate the Caspase-1/GSDMD pathway to induce pyroptosis [24]. The results showed that TAK1 was downregulated during the non-breeding season (*p* < 0.01), impairing its ability to inhibit the NLRP3 inflammasome and RIPK1. Activated RIPK1 can recruit FADD and pro-Caspase-8, leading to the activation of Caspase-8, which, in turn, cleaves GSDMD, promoting pyroptosis. As such, the transcription levels of *Casp-8*, *Ripk1*, *FADD*, and *Nlrp3* were upregulated during the non-breeding season. Research has found that, as Caspase-8 interacts with GSDMD by activating it, it also enhances TNF-induced lethality. GSDMD may also be cleaved by pathways independent of inflammasomes to induce antimicrobial effects. Meanwhile, Caspase-8 can interact with the pyroptosis adaptor protein ASC, with ASC-Caspase-8 leading, in some cases, to Caspase-8-dependent pyroptosis activation [42,43]. In addition, Caspase-8 can promote IL-1β activation and release by activating the NLRP3 inflammasome [44]. Additionally, Caspase-8 activation facilitates immune cell recruitment and fungal clearance, promoting the involvement of the Caspase-1/GSDMD signaling pathway in pyroptosis. The proper secretion of inflammatory factors such as IL-1β is crucial for limiting invading pathogens and effectively activating adaptive immune responses (Figure 5).

Overall, regulated pyroptosis is beneficial for the host’s defense [45]. In addition to pathogenic and inflammatory disease triggers, cytokines themselves can induce PANoptosis [46]. Caspase-8 and FADD are upstream regulators of NLRP3 inflammasome signaling, with the former being essential in both canonical and non-canonical NLRP3 inflammasome activation and playing a role in GSDMD-mediated IL-1β and IL-18 release and pyroptosis. Caspase-8 has a dual role in the transcriptional initiation and post-translational activation of both classical and non-classical pyroptosis pathways, revealing crosstalk between apoptosis and pyroptosis [47]. This indicates that the apoptosis and pyroptosis pathways are interrelated during the process of glandular atrophy. During the breeding season, muskrats are capable of secreting muskrat musk, which functions analogously to musk components. Owing to the scarcity of musk species, studying the secretion mechanism of muskrat musk glands can make up for the shortage of musk resources. Future research can further explore the dynamic changes in these apoptosis and pyroptosis-related genes at different developmental stages of the muskrats’ scented glands, as well as their specific mechanisms in regulating muskrats’ scented gland function. For example, the functions of these genes in scented gland cells can be specifically validated through gene knockout or overexpression techniques. At the same time, high-throughput sequencing can be used to systematically analyze the gene and protein expression profiles of fragrant glands at different developmental stages, thereby revealing more regulatory factors and signaling pathways.

## 5. Conclusions

The results of this study indicate that apoptosis drives the occurrence of muskrats’ scented gland atrophy, and there is a cross-over between GSDMD-based pyroptosis and apoptosis in this process, both of which play important regulatory roles simultaneously.

## Data Availability

The data presented in this study are available upon request from the corresponding author.

## Supplementary Materials

The following supporting information can be downloaded at https://www.mdpi.com/article/10.3390/ani14223194/s1: Table S1: Nucleotide sequences of primers for mRNA qPCR; Table S2: Transcriptome sequencing screening results.
